# An Extended Investigation of Unexpected *Helicoverpa zea* (Boddie) Survival and Ear Injury on a Transgenic Maize Hybrid Expressing Cry1A/Cry2A/Vip3A Toxins

**DOI:** 10.3390/toxins15070474

**Published:** 2023-07-22

**Authors:** Fangneng Huang, Ying Niu, Tiago Silva, Sebe Brown, Tyler Towles, Dawson Kerns, Juan Luis Jurat-Fuentes, Graham P. Head, Matthew Carroll, Wade Walker, Shucong Lin

**Affiliations:** 1 Department of Entomology, Louisiana State University Agricultural Center, Baton Rouge, LA 70803, USA; yniu@agcenter.lsu.edu (Y.N.); tsilva@agcenter.lsu.edu (T.S.); slin@agcenter.lsu.edu (S.L.); 2 Dean Lee Research & Extension Center, Louisiana State University Agricultural Center, Alexandria, LA 71302, USA; sbrow175@utk.edu (S.B.); wwalker@agcenter.lsu.edu (W.W.); 3 Macon Ridge Research Station, Louisiana State University Agricultural Center, Winnsboro, LA 71295, USA; tt305@msstate.edu; 4 Department of Entomology & Plant Pathology, University of Tennessee, Knoxville, TN 37996, USA; dkerns1@vols.utk.edu (D.K.); jurat@utk.edu (J.L.J.-F.); 5 Bayer Crop Science, St. Louis, MO 63167, USA; graham.head@bayer.com (G.P.H.); matthew.carroll1@bayer.com (M.C.)

**Keywords:** *Bt* maize, *Helicoverpa zea*, corn earworm, Cry, Vip3A, sentinel resistance, unexpected injury, resistance documentation

## Abstract

The wide occurrence of resistance to Cry1A and Cry2A insecticidal toxins from *Bacillus thuringiensis* (*Bt*) in the corn earworm/bollworm *Helicoverpa zea* (Boddie) leaves the Vip3A toxin produced during the vegetative stage of *Bt* as the only fully active toxin expressed in transgenic crops to control *H. zea* in the U.S.A. During 2021, the first unexpected survival of *H. zea* and injury (UXI) on a maize hybrid expressing Cry1A.105, Cry2Ab2, and Vip3Aa in Louisiana, U.S.A. were observed in two sentinel plots used for resistance monitoring. A follow-up intensive investigation was conducted with two *H. zea* populations established from larvae collected from the two UXI plots. The main goal of this study was to reveal if the unexpected damage was due to resistance development in the insect to the *Bt* toxins expressed in the maize hybrid. Diet-overlay bioassays showed that the two populations were highly resistant to Cry1A.105, moderately resistant to Cry2Ab2, but still highly susceptible to Vip3Aa when compared to a reference susceptible strain. In 10 d assays with detached ears, the larvae of the two UXI populations exhibited survival on ears expressing only Cry toxins but presented near 100% mortality on maize hybrids containing both *cry* and *vip3A* transgenes. Multiple field trials over three years demonstrated that natural *H. zea* populations in Louisiana were highly resistant to maize expressing only Cry toxins but remained susceptible to all tested hybrids containing *cry* and *vip3A* genes. Altogether, the results of this study suggest that the observed UXIs in Louisiana were associated with a resistance to Cry toxins but were not due to a resistance to Vip3A. The possible causes of the UXIs are discussed. The results generated and procedures adopted in this study help in determining thresholds for defining UXIs, assessing resistance risks, and documenting field resistance.

## 1. Introduction

Field populations of the corn earworm/bollworm *Helicoverpa zea* (Boddie) in the U.S.A. have developed resistance to Cry1A/Cry2A toxins expressed in transgenic maize and cotton producing insecticidal toxins from the bacterium *Bacillus thuringiensis* (*Bt*) [1,2,3,4,5,6,7]. To increase control efficacy and counteract Cry toxin resistance, *vip3Aa* genes (*vip3Aa19* for cotton and *vip3Aa20* for maize) have been incorporated into cotton and maize varieties that previously contained only *cry* transgenes for controlling lepidopteran pests [8]. Vip3Aa is an insecticidal toxin produced during the vegetative growth period of *Bt*, which has a distinct mode of action compared to Cry [9,10,11,12]. No cross-resistance between Vip3A and Cry toxins has been reported to date in resistant insects [4,13,14,15,16]. Moreover, *Bt* crop varieties containing *vip3A* genes have been effective in controlling *H. zea*, and to date, no practical resistance resulting in field control problems has been documented [4,5,17,18].

However, in areas where Cry1A/Cry2A resistance is widespread, Vip3A is the only consistently active toxin in all *Bt* crop varieties available to control *H. zea* [4]. Therefore, there is a great concern about the potentially rapid evolution of Vip3A resistance, especially in the southern region of the U.S.A. where both *Bt* maize and *Bt* cotton are planted, and *H. zea* is a cross-crop pest targeted by both *Bt* crops [19]. Although a resistance leading to control problems for Vip3A crop varieties has not been documented yet, the unexpected survival of *H. zea* and plant injury by the insect has been reported on several occasions, especially in cotton fields in the mid-southern states of the U.S.A. [17,20]. In addition, Vip3A resistance alleles have been detected in several field *H. zea* populations in the region [7,21], further demonstrating that the preservation of Vip3A susceptibility is critical for the sustainable use of *Bt* crop technology. 

In the U.S.A., planting sentinel plots for the resistance monitoring of *Bt* crops with *H. zea* is highly recommended [20,22]. A major goal of sentinel plot planting is monitoring unexpected pest survival or plant injury (UXI) to detect early signs of field-evolved resistance so that appropriate mitigation programs can be triggered to delay and/or contain the resistance to a limited area [22]. However, a common injury threshold to define a UXI is still lacking for *H. zea* on *Bt* maize containing *cry* and *vip3A* genes. As in other southern states of the U.S.A., *Bt* maize containing the *vip3A* gene has been planted in Louisiana for many years, but the area planted was very limited until recently [23]. Since 2017, Vip3A susceptibility in *H. zea* in Louisiana has been monitored annually by randomly checking >1000 ears of *Bt* plants at multiple locations across the state’s major maize planting areas. The field surveys showed rare insect survival on Vip3A maize, with most cases having 0 to 2 larvae (3rd instar or older) per 1000 ears (F. H., unpublished data). However, during the 2021 crop season, a greater than ‘normal’ *H. zea* survival and ear injury on a *Bt* maize hybrid containing *cry* and *vip3A* genes was observed in two sentinel plots at the Louisiana State University Agricultural Center’s (LSU AgCenter) Dean Lee Research Station in Alexandria, Louisiana. For convenience, hereafter we refer to this ‘greater than normal’ survival and ear injury as unexpected injury (UXI). The UXIs observed in 2021 represent the first UXIs reported for Vip3Aa maize traits in Louisiana.

Once a UXI is observed, it is important to document if it is caused by resistance development [20,23]. Thus, in this study, we conducted an extensive investigation including field insect collections, laboratory assays with purified *Bt* toxins and detached maize ears, and multiple field trials with common *Bt* maize traits (Table 1) to determine if the observed UXIs in Louisiana were due to the evolution of resistance to the *Bt* toxins in transgenic plants, especially to Vip3Aa. Our hypothesis was that, if the UXIs were due to resistance development, populations collected from the sentinel plants expressing Cry1A/Cry2A/Vip3A toxins should be resistant to the *Bt* toxins expressed in the plants. The results generated and procedures adopted in this study have important implications in determining thresholds for defining UXI, assessing resistance risk, and documenting field resistance.

## 2. Results

### 2.1. Unexpected H. zea Larval Survival and Ear Injury in 2021 in Two Sentinel Plots in Louisiana

On 16 June 2021, unexpected *H. zea* larval survival and ear injury (UXI) were observed in two sentinel plots on a *Bt* maize hybrid (DKC 65-99) expressing the Cry1A.105/Cry2Ab2/Vip3Aa20 *Bt* toxins (hereafter referred as SNT-I and SNT-II) at the LSU AgCenter’s Dean Lee Research Station near Alexandria, Louisiana, U.S.A. (Table S1). Field surveys on the next day showed that 2.5% of the ears in SNT-I were injured, and 2.1% of the ears contained live *H. zea* larvae with an ear injury area of 2.3 cm^2^ per ear (Table S1). Similar observations were recorded in SNT-II, with 3.7% ears injured, 3.5% ears containing larvae, and an injury of 2.2 cm^2^ per ear. Most larvae observed in the UXIs were third instars (58.5% of the total), followed by second instars (33.3%) and fourth instars (8.1%). 

In the same two sentinel plots, no unexpected insect survival and plant injury were observed on two other *Bt* maize hybrids: NK 1694-3111 expressing Cry1Ab and Vip3Aa, and PI 1622 VYHR expressing the Cry1Ab, Cry1F, and Vip3Aa toxins. In contrast, 62.3% of ears of a non-*Bt* corn hybrid (DKC 65-93) were injured by *H. zea*, and 26.2% of the ears contained live larvae with an injury of 3.8 cm^2^ per ear. The non-*Bt* maize hybrid was planted adjacent to the DKC 65-99 plot at the same time in SNT-I. The larvae observed on the non-*Bt* maize ears consisted of second (37.5% of total), third (18.8%), fourth (18.8%), fifth (18.8%), and sixth (6.3%) instars (Table S1). In addition, ELISA-based assays (EnviroLogix, Quantiplate^TM^ kits, Portland, ME, U.S.A.) confirmed Cry1A/Cry2A, and Vip3Aa toxin expression in all ten randomly sampled DKC 65-99 plants, while no expression of *Bt* toxins was detected in ten non-*Bt* plants. 

### 2.2. Field-Derived Populations of H. zea Collected from SNT-I and SNT-II Were Resistant to Cry1A.105 and Cry2Ab2, but Still Susceptible to Vip3Aa

Relative to a reference susceptible *H. zea* laboratory strain (BZ), diet-overlay bioassays [20] showed that the two populations (UXI_LA1_ and UXI_LA2_) collected from the ears of plants expressing Cry1A.105, Cry2Ab2, and Vip3Aa in SNT-I and SNT -II were highly resistant to Cry1A.105, with resistance ratios of 234- and 134-fold for each strain, respectively (Table 2). The two populations also exhibited a moderate level (5-fold) of resistance to Cry2Ab2. In contrast, both populations were susceptible to Vip3Aa as the reference (BZ) strain. Moreover, nonoverlapping 95% confidence limits supported that the LC_50_ values of UXI_LA1_ (0.265 µg/cm^2^) and UXI_LA2_ (0.016 µg/cm^2^) for Vip3Aa were significantly lower than the LC_50_ (0.451 µg/cm_2_) for BZ (Table 2). In addition, for each of the three *Bt* toxins tested, the overall bioassay dose-responses of UXI_LA1_ and UXI_LA2_ were similar to the responses of two field-derived populations collected from non-*Bt* maize: (1) NBT_DL_ collected at the same location as UXI_LA1,_ and (2) NBT_SG_ from southern Louisiana where maize is not a major crop (Table 2).

### 2.3. Maize Expressing Cry1A/Cry2A Was Partially Active against UXI_LA1_ and UXI_LA2_, While Larval Mortality on Ears Containing Cry and vip3A Transgenes Was Nearly 100%

**Figure 1:**
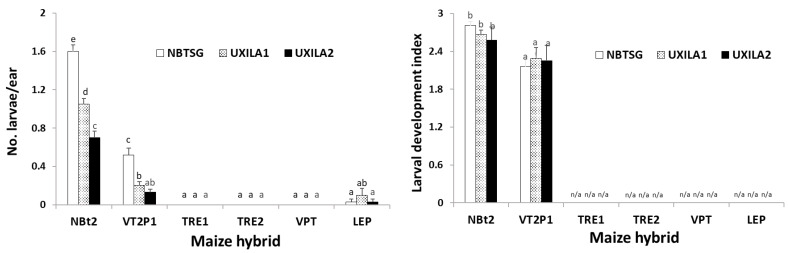
Larval survival and development index of *Helicoverpa zea* populations from UXI plots on detached ears of non-*Bt* and *Bt* maize hybrids. Mean values within a figure followed by the same letter are not significantly different (Tukey HSD test, α = 0.05). n/a: data not available due to no or few larvae recovered.

An analysis of variance (ANOVA) on the data from detached ear assays with one non-*Bt* (NBt2) and five *Bt* hybrids (VT2P1, TRE1, TRE2, VPT, and LEP) showed that the effects of insect population and the maize hybrids on 10 d larval survival were significant (*F*_2,51_ = 34.15; *p* < 0.0001 for insect population and *F*_5,51_ = 395.19; *p* < 0.0001 for the maize hybrids), as well as for their interaction (*F*_10,51_ = 15.48; *p* < 0.0001). On non-*Bt* (NBt2) ears that were originally infested with four *H. zea* neonates/ear, an average of 1.60, 1.05, and 0.70 larvae per ear of NBT_SG_, UXI_LA1_, and UXI_LA2_ survived after 10 d, respectively (Figure 1). After the same period, 0.52, 0.20, and 0.13 larvae of the three populations, respectively, survived on the hybrid (VT2P1) expressing Cry1A.105 and Cry2Ab2, which was significantly less (*p* < 0.05) than the mortality observed on the non-*Bt* hybrids (Figure 1). In contrast, 100% of larvae from the three insect populations were killed on three of the four hybrids expressing both Cry and Vip3Aa toxins (TRE1, TRE2, and VPT). On the hybrid (LEP) expressing Cry1Ab, Cry1F, and Vip3Aa, a total of 5 larvae (1 NBT_SG_, 3 UXI_LA1_, and 1 UXI_LA2_) survived from the total of 480 neonates infested on 120 ears. The number of surviving larvae observed on LEP was not significantly (*p* > 0.05) different from zero (Figure 1). 

Due to the lack of sufficient *H. zea* larvae recovered from the three traits expressing Cry and Vip3Aa toxins, larval development data for the abovementioned five live larvae on LEP were excluded in the ANOVA. The analysis of larval development data observed on NBt2 and VT2P1 showed that the effect of the maize hybrids on larval development was significant (*F*_2,16_ = 6.68; *p* = 0.0078), while it was not significant for insects (*F*_2,16_ = 0.13; *p* = 0.8773) and their interaction (*F*_2,16_ = 0.49; *p* = 0.6206). Most of the larvae recovered from non-*Bt* ears were third instars. Compared to non-*Bt* ears, the larval development on VT2P1 was significantly (*p <* 0.05) delayed. In addition, four of the five live larvae recovered from LEP were still second instars, and the other was a third instar. 

### 2.4. Maize Expressing Only Cry Toxins Failed to Control H. zea in Multiple Field Trials, While Hybrids Expressing Cry and Vip3Aa Toxins Were Still Highly Effective, including in the Area Where the UXIs Were Observed

**Figure 2:**
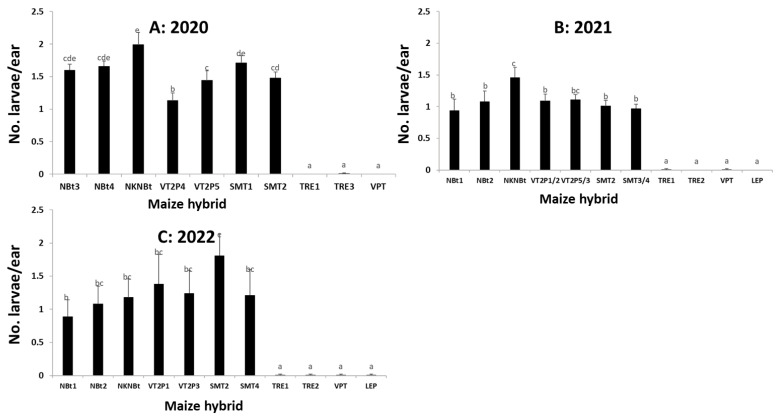
Larval occurrence of *Helicoverpa zea* on non-*Bt* and *Bt* maize hybrids in field trials in 2020 (**A**), 2021 (**B**), and 2022 (**C**). There were two trials in each year, and the data from the two trials were pooled in an ANOVA for each year. Mean values within a figure followed by the same letter are not significantly different (Tukey HSD test, α = 0.05).

Nine field trials from 2020 to 2022 (two, Trial-I and Trial-II, in 2020; two, Trial-III and Trial-IV, in 2021; and five, Trial-V to -IX, in 2022) consistently showed that maize expressing only Cry toxins failed to control *H. zea*, while hybrids expressing Cry and Vip3Aa toxins were still highly effective, including in the area where the UXIs were observed. An ANOVA based on the two trials in each year (Trial-I and -II in 2020, Trial-III and -IV in 2021, and Trail-V and -IV in 2022) that recorded larval occurrence showed that the effects of the maize hybrids on larval occurrence were significant for each of the six individual trials (Tables S3–S5), as well as for a pooled data analysis for each of the three years (*F*_9,57_ = 147.19, *p* < 0.0001 for 2020; *F*_10,70_ = 52.26, *p* < 0.0001 for 2021; and *F*_10,70_ = 17.21, *p* < 0.0001 for 2022) (Figure 2). The larval occurrence on the three non-*Bt* hybrids ranged from 1.60 to 1.99 larvae/ear for the trials in 2020, 0.94 to 1.46 in 2021, and 0.89 to 1.18 in 2022 (Figure 2). The larval occurrence was not significantly (*p* > 0.05) different among the three non-*Bt* hybrids evaluated in 2020 and 2022, while the occurrence (1.46 larvae/ear) observed in 2021 on non-*Bt* NKNBt was greater (*p* < 0.05) than the numbers observed on the other two non-*Bt* hybrids (Figure 2). 

On *Bt* plants expressing Cry1A.105, Cry2Ab2, and Cry1F only, for the two trials in 2020, an average of 1.71 larvae/ear was observed on SMT1, which was not significantly (*p* > 0.05) different from the occurrence on the three non-*Bt* hybrids evaluated that year. However, the larval occurrence on SMT1 was greater (*p* < 0.05) than the occurrence (1.33 to 1.48 larvae/ear) observed on the other three hybrids (SMT2, VT2P4, and VT2P5) (Figure 2). For the trials in 2021 and 2022, the larval occurrence among the hybrids expressing only Cry toxins (SMT and VT2P) was similar (*p* > 0.05) for each of the two years, ranging from 0.97 to 1.11 larvae/ear in 2021 and 1.21 to 1.81 in 2022 (Figure 2). In contrast, all hybrids expressing Cry and Vip3Aa toxins, including DKC 65-99 (TRE1), that were associated with the observed UXIs, were extremely effective against field populations of *H. zea*, with only seven live larvae recovered from 880 ears sampled across the six trials and in the three years (Figure 2). 

**Figure 3:**
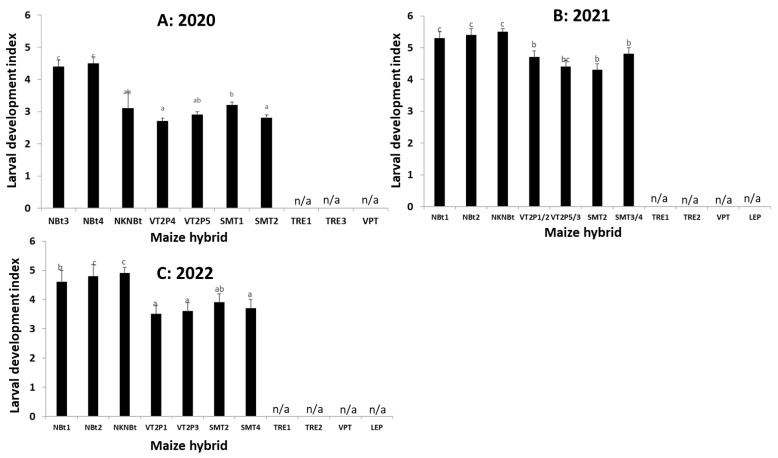
Larval development index of *Helicoverpa zea* on non-*Bt* and *Bt* maize hybrids in field trials in 2020 (**A**), 2021 (**B**), and 2022 (**C**). There were two trials in each year, and the data from the two trials were pooled in an ANOVA for each year. Mean values within a figure followed by the same letter are not significantly different (Tukey HSD test, α = 0.05). n/a: data not available due to no or few larvae recovered.

Due to the lack of sufficient larvae being recovered from the hybrids expressing Cry and Vip3Aa toxins, the developmental data on these seven larvae were excluded in ANOVA analyses. An analysis of the data collected from non-*Bt* and *Bt* hybrids expressing Cry toxins only showed that the effect of maize on larval development was significant for each individual trial during the three years except Trial-VI in 2022 (Tables S3–S5), and the effect was significant for each of the three years in the pooled data analysis (*F*_6,36_ = 28.32, *p* < 0.0001 for 2020; *F*_6,42_ = 12.30, *p* < 0.0001 for 2021; and *F*_6,42_ = 4.79, *p* = 0.0008 for 2022) (Figure 3). In general, the larval development on *Bt* maize expressing only Cry toxins (e.g., VT2P and SMT) was delayed by approximately one instar relative to the larvae recovered from non-*Bt* ears, with few exceptions (Figure 3). In addition, the seven live larvae observed from maize containing *cry* and *vip3Aa* genes consisted of one second, three third, and three fourth instars. 

**Figure 4:**
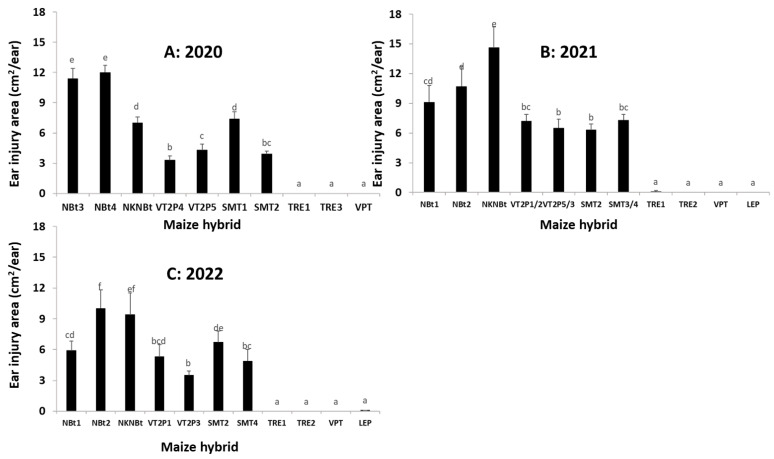
Ear injury area by *Helicoverpa zea* on non-*Bt* and *Bt* maize hybrids in field trials in 2020 (**A**), 2021 (**B**), and 2022 (**C**). There were two trials in each year, and the data from the two trials were pooled in an ANOVA for each year. Mean values within a figure followed by the same letter are not significantly different (Tukey HSD test, α = 0.05).

**Figure 5:**
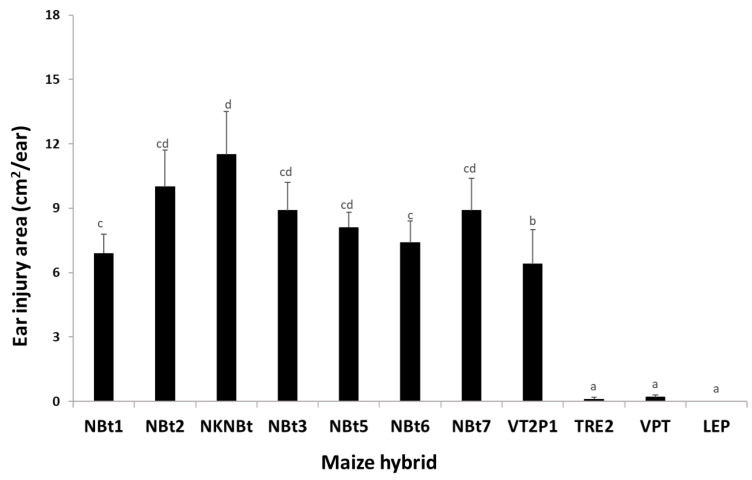
Ear injury area by *Helicoverpa zea* on non-*Bt* and *Bt* maize hybrids in three additional trials (Trial-VII to Trial-IX) in 2022. The data from the three trials were pooled in an ANOVA. Mean values in the figure followed by the same letter are not significantly different (Tukey HSD test, α = 0.05).

Besides the abovementioned six trials (Trial-I to -VI), three additional trials (Trial-VII, -VIII, and -IX) evaluated ear injury by *H. zea* only. An ANOVA for all the nine trials during the three years showed that the effects of the maize hybrids on ear injury were all significant for each individual trial (Tables S3–S6), as well as for the pooled data analysis for each of the three years (*F*_9,57_ = 202.26, *p* < 0.0001 for 2020; *F*_10,70_ = 190.03, *p* < 0.0001 for 2021; *F*_10,70_ = 104.05, *p* < 0.0001 for the pooled data of Trial-V and -VI in 2022; *F*_10,110_ = 100.37, *p* < 0.0001 for the combined data of Trial-VII to IX in 2022). Ear injury areas of non-*Bt* hybrids ranged from 7.0 to 12.0 cm^2^/ear with an average of 10.1 for the trials in 2020, 9.1 to 14.6 with an average of 11.5 in 2021, 5.9 to 10.0 with an average of 8.4 for Trial-V and -VI in 2022, and 6.9 to 11.5 with an average of 8.8 for Trial-VII to -IX in 2022 (Figure 4 and Figure 5). Notable ear injuries by *H. zea* were also observed for all hybrids expressing only Cry toxins, but the injury areas of these hybrids were generally smaller (*p* < 0.05) than observed on non-*Bt* ears. In contrast, hybrids expressing Cry and Vip3Aa toxins were virtually free from injury by *H. zea* (Figure 4 and Figure 5, Tables S3–S6). 

### 2.5. Relative to Non-Bt Maize, H. zea Occurrence Rate and Reduction on Ear Injury in Field Trials Were Similar from 2018 to 2022 for Each Bt Maize Trait

**Figure 6:**
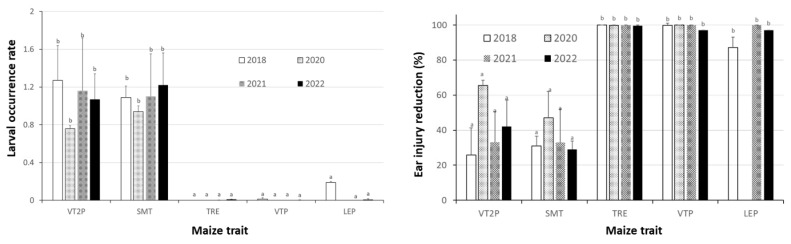
Relative insect occurrence rate and ear injury reduction (%) of five common *Bt* maize traits in 11 field trials in Louisiana from 2018 to 2022. The relative insect occurrence rate on a *Bt* maize trait in a trial was calculated by dividing the larval occurrence on the *Bt* trait divided by the mean occurrence on the non-*Bt* traits in the trial as follows: % reduction in ear injury = 100 × (injury area on non-*Bt* ears—injury area on *Bt* ears)/injury area on non-*Bt* area. Mean values in the figure followed by the same letter are not significantly different (Tukey HSD test, α = 0.05).

A two-way ANOVA with data generated from the nine trials from 2020 to 2022, together with the data of two similar field trials in 2018 [4], showed that the main effects of the maize trait on the relative occurrence and injury reduction parameters were significant (*F*_4,19_ = 28.14, *p* < 0.0001 and *F*_4,31_ = 57.71, *p* < 0.0001, respectively). However, the effects of the ‘year’ were not significant (*F*_3,19_ = 0.57, *p* = 0.6404 for relative occurrence and *F*_3,31_ = 1.71, *p* =0.1863 for injury reduction), and the interaction of the two factors was also not significant (*F*_11,19_ = 0.26, *p* = 0.9878 for relative occurrence and *F*_11,31_ = 0.68, *p* = 0.7455 for injury reduction). During the five-year period, the relative occurrence rates of the two traits (VT2P and SMT) expressing Cry toxins ranged from 0.76 to 1.27 and from 0.94 to 1.22, respectively. The differences in the relative occurrence among years and between the two traits were not significant (*p* > 0.05), with an average of 1.07 across the five years for both traits (Figure 6). However, the two traits still reduced (*p* < 0.05) ear injury by 35.0 to 41.8%, and the reductions were similar across years and between the two traits. 

In contrast, the relative larval occurrence rates on the three traits (TRE, LEP, and VPT) containing *cry* and *vip3Aa* genes were zero or close to zero, and the ear injuries were reduced by 99.3 to 99.7%. The high effectiveness of the three traits against the field *H. zea* populations were consistent (*p* > 0.05) across the five years from 2018 to 2022 (Figure 6). In addition, a regression analysis showed that there were no significant trends in the relative larval occurrence rate and injury reduction data for each of the five *Bt* maize traits (across the five years, *t* values ranging from 0.20 to 3.72 and *p* values ranging from 0.1673 to 0.8603).

## 3. Discussion

To date, practical (or field) resistance to *Bt* crops resulting in field control problems has been clearly documented in more than 20 cases [24]. Although the specific approaches for resistance documentation vary, there are two steps that are commonly involved for all cases: (1) the occurrence of unexpected control problems in the field and (2) follow-up confirmation that the field control problem is due to resistance development [23]. In this regard, the results herein of the follow-up investigations suggest that the observed UXIs in Louisiana were associated with a resistance to Cry toxins, yet the results did not provide evidence supporting that the UXIs were due to a resistance development to Vip3Aa. The observed UXIs not being associated with a resistance to Vip3Aa is not an exception. An earlier study in [25] also did not provide evidence to conclude that a UXI of a *Bt* maize hybrid caused by the sugarcane borer *Diatraea saccharalis* (F.) in 2009 in Louisiana was due to a resistance to the *Bt* toxins in the plants. In fact, our follow-up long-term extended monitoring shows that to date, *D. saccharalis* has remained susceptible to *Bt* maize, and no signs of resistance have been observed. In addition, an early study [17] reported that an *H. zea* population collected in Texas in 2018 from a UXI on a maize hybrid expressing *cry1F*, *cry1Ab*, and *Vip3Aa20* transgenes had a low level of reduced susceptibility to Vip3Aa51, >94.8% identical to Vip3Aa20, yet to date no practical resistance or additional field control problems with Vip3Aa maize traits have been reported in the area. 

We understand that data generated from the current study could not completely exclude resistance development to Vip3Aa being a cause of the observed UXIs in Louisiana. However, we believe that several other factors and their combinations might have played a more important role in the UXIs in the current study. First, as documented in the current study, a resistance to Cry1A and Cry2A toxins in *Bt* maize is widely detected for *H. zea* in Louisiana, including the location where the UXIs were observed [4,6,16]. Earlier studies reported that maize hybrids expressing pyramided Cry and Vip3Aa toxins were very effective [26] and likely expressed a pyramided ‘high dose’ (or very close to be considered a ‘high dose’) against *H. zea* [27]. However, once the Cry toxins lost/reduced their activities, the maize containing *cry* and *vip3Aa* genes would function as a single-gene *Bt* trait [4] so that the plants no longer produced a pyramided ‘high dose’ against the Cry-toxin-resistant *H. zea* [22]. Second, both UXIs observed in this study occurred in sentinel plots, one in an 8-row plot and another consisting of 16 rows. Since non-*Bt* and other *Bt* traits were also planted in the same sentinel fields, pollen drift between plots could reduce Vip3Aa expression in the plants and diminish its effectiveness [28]. In fact, a notable similarity among the UXIs associated with *D. saccharalis* reported in 2009 [25], the UXIs with *H. zea* in Texas in 2018 [17], and the current UXIs with *H. zea* in Louisiana is that they were all observed in field trial plots, while UXIs for most documented cases summarized in [24] were originally reported in grower production fields. If pollen drift was indeed a factor contributing to the observed UXIs, rational plot arrangements that should avoid such false positives are necessary in planting sentinels for resistance monitoring.

In addition, several studies have shown that the expression of *Bt* transgenes in plants is affected by environmental factors, especially under some stress conditions affecting the primary and secondary metabolism of *Bt* plants [29]. For example, Trtikova et al. [30] reported that the expression of the *cry1Ab* transgene in MON 810 maize could be reduced under hot/dry stressed environments compared to optimal conditions. Recently, Liu et al. [31] also observed that Cry1Ab/c expression in transgenic cotton plants varied in different planting environments. Thus, a reduction in Vip3Aa expression under severe stress conditions coupled with a resistance to Cry toxins might have caused the observed UXIs in the sentinel plots. 

To date, genes that allow resistant *H. zea* individuals to survive and complete their life cycle on commercial Vip3Aa maize have not been documented, but alleles showing high levels of resistance to the toxin have been reported in several field-collected populations [7,17]. In addition, functional minor resistance alleles to Vip3Aa have been reported in *H. zea* [16]. The relatively high rate of individuals carrying such minor resistance alleles might also enhance the survival of the field populations to a level observed in the UXIs.

Furthermore, the two UXIs observed in this study were not independent from each other. Both UXIs were associated with the same hybrid, while other hybrids containing the *vip3Aa* gene in the same sentinel fields did not have any control problems. Both sentinel plots were planted on the same day, and the distance between the two plots was only ~500 m, thus any factors affecting the performance of the hybrid would occur in both sentinel fields. 

Although this study did not provide evidence of a resistance to Vip3Aa in the field, the occurrence of the observed UXIs still could be an indicator of a potential risk for resistance development to the toxin if effective IRM plans preserving Vip3Aa susceptibility are not implemented. More importantly, the observed UXIs strongly suggest that Vip3Aa expression alone in *Bt* maize is not a ‘high dose’ for *H. zea*, and its control efficacy could be further diminished by environmental factors. The reduced toxin protein expression in a nonhigh dose system could enhance the survival of resistant heterozygotes and/or individuals possessing minor resistance alleles, thus increasing the risk of resistance development.

## 4. Conclusions

Two UXIs caused by *H. zea* on a maize hybrid expressing Cry1A.105, Cry2Ab2, and Vip3Aa toxins were observed in two sentinel plots for resistance monitoring in Louisiana (U.S.A.) in 2021. Diet-overlay bioassays showed that the two populations of *H. zea* collected from the two UXI plots (UXI_LA1_ and UXI_LA2_) were resistant to Cry1A.105 and Cry2Ab2, but still susceptible to Vip3Aa. Laboratory assays with detached maize ears from hybrids expressing only Cry toxins were partially active against UXI_LA1_ and UXI_LA2_, while a nearly 100% larval mortality of the two populations was observed after 10 days on maize expressing Cry and Vip3Aa. In field trials, maize expressing only Cry toxins failed to control *H. zea*, while hybrids expressing Cry and Vip3Aa toxins were highly effective against the insect, including in the area where UXIs were observed. Relative to non-*Bt* maize, *H. zea* occurrence rate and ear injury reduction on each of the five *Bt* maize traits tested in the field were similar from 2018 to 2022. Altogether, the results of this study suggest that the observed UXIs in the two sentinel plots in Louisiana were associated with a resistance to Cry toxins, but the study does not provide evidence to support that the UXIs were due to a resistance development to Vip3Aa. Possible causes of the observed UXIs include (1) the common occurrence of resistance to Cry toxins in *H. zea*, (2) a reduced *Bt* protein expression and control efficacy due to pollen drift between the plots of the sentinel plantings, (3) a reduced Vip3Aa expression due to some environmental stress factors, and (4) the existence of low-level Vip3Aa resistance alleles. Nevertheless, the observed UXIs in this study are still a clear sign of the potential risk for resistance development to Vip3Aa in maize if effective IRM plans are not implemented. In addition, the data generated and the procedures adopted in this study have value in defining UXIs of *H. zea* on Vip3Aa maize, arranging field plots in sentinel planting, and in resistance documentation. 

## 5. Materials and Methods

### 5.1. Field Surveys of H. zea Occurrence and Ear Injury in Sentinel Plots for Bt Resistance Monitoring in LOUISIANA 

Two UXIs of maize hybrid DKC 65-99 containing the Trecepta^®^ (TRE) trait expressing Cry1A.105, Cry2Ab2, and Vip3Aa toxins were observed in the 2021 crop season in two sentinel plots (SNT-I and SNT-II) set for resistance monitoring in Louisiana, U.S.A. (Table S1). The two sentinel plots were planted at the same time, and both consisted of a single replication with non-*Bt* and three *Bt* maize hybrids representing three traits: (1) TRE, (2) Agrisure Viptera^TM^ (VPT) containing Cry1Ab and Vip3Aa, and (3) Leptra^TM^ (LEP) containing Cry1Ab, Cry1F, and Vip3Aa (Table S1). Each plot in SNT-I for a hybrid was 8-rows wide and approximately 25 m long, and the plot size for SNT-II was 16 rows and about 33 m long. The distance between the two SNTs was ca. 500 m. Field surveys were performed the day after the UXIs were identified. In the surveys, a total of 4105 maize ears were checked from the two plots (1663 ears from SNT-I and 2442 from SNT-II), and the number of live *H. zea* larvae, larval stages, and ear injury area were recorded for each ear. The toxin protein expression of Cry1A/Cry2A, and Vip3Aa toxins in the leaf tissue of 10 randomly sampled DKC 65-99 TRE plants and 10 non-*Bt* plants was assessed by ELISA-based assays (EnviroLogix, Quantiplate^TM^ kits, Portland, ME, U.S.A.).

### 5.2. Insect Collections and Establishment of Field-Derived H. zea Populations in the Laboratory 

To document if the UXIs observed in the sentinel plots were related to resistance development, four populations of *H. zea* (named UXI_LA1_, UXI_LA2_, NBT_DL_, and NBT_SG_) were collected from maize fields in Louisiana. UXI_LA1_ and UXI_LA2_ were established with larvae collected from the ears of DKC 65-99 in SNT-I and SNT-2, respectively. Because the larval development on the ears in the UXI plots varied from 2nd to 4th instars (Table S1), the 3rd and 4th instars collected for each UXI plot during the field surveys were directly reared in 30 mL plastic cups (Fill-Rite, Newark, NJ, U.S.A.) containing a meridic diet (Ward’s Stonefly Heliothis diet, Rochester, NY, U.S.A.), as described in [4]. If a larva on the ear was still at the 2nd instar, the ear containing the young larva was removed from the plant, brought to the lab, and then placed in a 5.7 L plastic box (2 ears/box; 32 cm long × 19 cm wide × 12 cm high) for continued rearing in the laboratory until the larva developed to the 3rd or 4th instar. The larvae that developed to the 3rd or 4th instar were then transferred individually to the 30 mL plastic cups containing the same meridic diet, and then were placed together with the field-collected larvae for each UXI plot. During the larval rearing and pupation, varied temperatures were used to synchronize the development of the insect populations. At the end, a total of 55 pupae were available to establish UXI_LA1_, and 63 pupae were used to generate UXI_LA2_ (Table S2). NBT_DL_ was established from 87 larvae (3rd to 6th instars) collected during the field survey from a non-*Bt* hybrid (DKC 65-93) in SNT-I. Another population, NBT_SG,_ was originated from 132 larvae (3rd to 6th instars) collected from non-*Bt* maize at LSU AgCenter’s research station near St. Gabriel in southern Louisiana (Table S2). 

Pupae that developed from the diet rearing in the laboratory were collected for each population and placed in 20 L mesh cages (Seville Classics, INC., Torrance, CA, U.S.A.) containing vermiculite (Sun Gro, Pine Bluff, AR, U.S.A.) and 10% honey water solution. The cages containing pupae were arranged in an insect-rearing room at ~26 °C, >70% RH, with a 14:10 h (L:D) photoperiod for adult emergence, mating, and oviposition [4]. The F1 or F2 neonates produced from the field-collected populations were used for this study (Table S2). In addition to the four field-derived populations, a laboratory strain (BZ) of *H. zea* was also included as a reference in the study. The BZ strain was obtained from Benzon Research Inc. (Carlisle, PA, U.S.A.) and had been reared in the laboratory for many generations without exposure to any *Bt* toxins or chemical insecticides. This BZ strain has been documented to be susceptible to Cry1A.105, Cry2Ab2, Vip3Aa, and corn plants expressing one or more of these toxins [6,16]. 

### 5.3. Sources of Bt Toxins for Diet-Overlay Bioassays 

The susceptibility of BZ to three individual *Bt* toxins (Cry1A.105, Cry2Ab2, and Vip3Aa39) and the four insect populations of *H. zea* that originated from the field collections was determined using a diet-overlay bioassay method [4]. The *Bt* toxins used in these bioassays are the same or closely related to the corresponding *Bt* toxins expressed in the TRE maize hybrid associated with the observed UXIs. The two Cry toxins expressed in the hybrid with the UXIs, Cry1A.105 and Cry2Ab2, were provided by Bayer Crop Science (St. Louis, MO, U.S.A.). The Vip3Aa39 toxin was provided by the Insect Molecular Pathology and Resistance (IMPaR) laboratory at the University of Tennessee (Knoxville, TN, U.S.A.). The Vip3Aa20 toxin expressed in maize shares the same similarity (94.8%) with Vip3Aa19 expressed in cotton and the Vip3Aa39 toxin used in our bioassays [32], supporting the same mode of action. Information on the toxin production in *E. coli* cultures and purification was described in [6] for Cry1A.105 and Cry2Ab2, and in [21] for Vip3Aa39. 

### 5.4. Diet-Overlay Bioassays to Determine the Susceptibility of H. zea Populations to Cry1A.105, Cry2Ab2, and Vip3Aa Toxins

The diet-overlay bioassay method used in this study was similar to that described in [4,16]. Briefly, *Bt* toxin solutions were prepared in distilled water containing 0.1% Triton X-100, and 50 µL was dispensed on individual wells of 128-cell CD-International bioassay trays (Pitman, NJ, U.S.A.) containing approximately 0.8 mL of Southland diet (Lake Village, AR, U.S.A.) in each cell. After the solution on the diet surface was dry, one neonate (<24 h old) was inoculated per well. Each bioassay consisted of 7 to 8 *Bt* toxin concentrations ranging from 0.00316 to 10.0 µg/cm^2^, as well as a negative control that was treated with the corresponding *Bt* toxin buffer and a blank control that was treated with distilled water containing 0.1% Triton X-100. In each bioassay, there were four replications for each *Bt* concentration and control, and each replication consisted of 16 to 32 larvae. Bioassay trays were placed in a growth chamber and maintained at 26 °C, ~50% RH, with a photoperiod of 16:8 h (L:D). The number of dead and live larvae that were severely stunted and still at the 1st or 2nd instar were recorded 7 days after neonate inoculation.

Larval mortality in the bioassays was measured as ‘practical mortality’, which considered both dead larvae and the severely stunted living larvae (still at the 1st or 2nd instars after the 7 days bioassay) considered ‘practically dead’ [6]. Larval ‘practical mortality’ at a *Bt* concentration was corrected using the control mortality, and the corrected mortalities were analyzed with probit analysis [33] using SAS PROC PROBIT [34] to determine the medium lethal concentration (LC_50_) that resulted in 50% larval mortality and the associated 95% confidence interval. The resistance ratio of field-collected populations was calculated based on their LC_50_ values for each *Bt* toxin compared to the BZ strain [6]. 

### 5.5. Detached Ear Assays to Determine the Survival and Development of H. zea Populations on Non-Bt and Bt Maize Expressing Only Cry and/or Vip3Aa Toxins

To validate if the field *H. zea* populations collected from the UXI plots were functionally resistant to *Bt* maize containing *cry* and *vip3Aa* genes, larval survival and the development of NBT_SG,_ UXI_LA1_, and UXI_LA2_ were evaluated on the detached ears of a non-*Bt* and five *Bt* maize hybrids in the laboratory (Figure 1). During the 2021 crop season, maize seeds were hand-planted in a single-row field plot (~350 ft long) for each hybrid at the LSU AgCenter’s Research Station in Winnsboro, Louisiana. There was a two-row alley between maize traits to minimize cross-pollination. Toxin protein expression in the *Bt* maize hybrids and nonexpression in the non-*Bt* hybrid were confirmed using ELISA test strips as described above. When the field plants developed to the late R1 stage, ears along with ear silks, sheaths, and husks were removed from the plants. The field-collected ears were brought to the laboratory and carefully inspected to remove any natural *H. zea* larvae before the ears were used in the study. 

The larval survival and growth of UXI_LA1_, UXI_LA2_, and NBT_SG_ on the five maize hybrids were evaluated using a detached-ear assay method. In the laboratory, four neonates (<24 h old) were placed on the silks of each detached ear, and the infested ears were placed in 5.7 L plastic boxes (5 ears/box). The plastic boxes containing infested ears were placed in a walk-in insect-rearing room maintained at 24 °C, ca. 50% RH, with a photoperiod of 16:8 h (L:D). The detached-ear assays in the room were arranged in a randomized complete block (RCB) design with four replications for each combination of insect population and maize hybrid with 10 ears (or 40 neonates in two boxes) in each replication. To facilitate the RCB design, the insect-rearing room was divided into four sections, and each section represented a block. The number of live larvae and larval developmental stages on each ear were recorded 10 d after neonate inoculation. 

The data on larval development stages (instars) were converted to a ‘larval development index’: 1 = 1st instar, 2 = 2nd instar, …, 6 = 6th instar, and 7 = pupal stage. The data on the number of living larvae and larval development index were transformed with log (x + 1) for normality. Transformed data were then analyzed using a two-way analysis of variance (ANOVA) with the maize hybrid (trait) and insect population as the two main factors (SAS PROC GLM) [34]. Treatment means were separated using Tukey’s HSD at α = 0.05. 

### 5.6. Field Trials to Monitor the Occurrence of H. zea and Ear Injury on Common Bt Maize Traits

From 2020 to 2022, nine trials (Trial-I to -IX) were conducted to monitor the occurrence of *H. zea* and the associated ear injury in three locations in Louisiana (Figure 2, Figure 3, Figure 4 and Figure 5, Tables S4–S7). Trial-I, -III, -V, and -VII were performed at the LSU AgCenter’s Dean Lee Research Station in central Louisiana, near Alexandria, where the UXIs were observed. Trial-II, -IV, VI, and VIII were conducted at the LSU AgCenter’s Macon Ridge Research Station in northeast Louisiana near Winnsboro. Trial-IX was located at the LSU AgCenter’s Northeast Research Station near St. Joseph. The straight-line distance is ca. 120 km between the Dean Lee and Macon Ridge stations, and 138 km between the Dean Lee and Northeast stations. 

Each of Trial-I to -VI consisted of three non-*Bt* and seven or eight *Bt* maize hybrids (Tables S3–S5). The *Bt* hybrids in these trials represented five common *Bt* maize traits including Genuity^®^VT Double PRO^TM^ (VT2P), Genuity^®^ SmartStax^®^ (SMT), TRE, VPT, and LEP (Table 1). VT2P contains both *cry1A.105* and *cry2Ab2* genes for controlling lepidopteran moth pests, and SMT possesses the same two *cry* genes plus *cry1F* (Table S1) [8]. Each of Trial-VII, -VIII, and -IX tested five non-*Bt* and three *Bt* maize hybrids (Figure 6, Table S6). The three *Bt* hybrids contained TRE, VPT, and LEP, respectively. The field plots of each trial were 4-rows wide and 7.62 to 9.14 m long, and each trial was arranged in a randomized complete block design with four replications. For Trial-I to -VI, the larval occurrence and development stages of *H. zea* and the ear injury area caused by the insect were recorded by randomly sampling 20 ears in the two central rows (10 ears/row) for each plot when most of the larvae reached the 3rd to 5th instar, depending on trials. For Trial-VII to -IX, field sampling was performed at somewhat later plant stages to maximize ear injury levels, and thus only ear injury data were recorded by randomly sampling 25 ears from the central two rows in each plot. The expression of *Bt* toxins in the tested hybrids was confirmed using the ELISA test strips described above. 

In maize fields, the mature larvae of *H. zea* usually move out from the ears and then drop into the soil for pupation. Thus, the larval occurrence data on maize ears in Trial-I to Trial-VI were adjusted using a similar method as described in [18]. Briefly, one 6th instar was added to the data if an ear with no accountable larvae was observed but had an ear injury area of >6 but ≤12 cm^2^, two 6th instars were added to the data if the ear injury area of an ear was greater than 12 cm^2^ without accountable larvae, while one 6th instar was added for an ear with an ear injury area of >12 cm^2^ and only one accountable larva. No adjustments were made for any other cases. The data on the adjusted number of larvae and ear injury area were transformed with log (x + 1) for normality, and the transformed data were then analyzed with a one-way ANOVA (SAS PROC GLM) [34] for each trial with the maize hybrid as the main factor. To increase the degrees of freedom in the ANOVA, Trial-I to Trial-VI data generated from the two trials in a year as well as the data from Trial-VII to Trial-IX in 2022 were pooled, and the pooled data were further analyzed with a mixed model ANOVA with the ‘trial’ as a random factor (SAS PROC MIXED) [34]. The treatment means in all ANOVAs were separated using Tukey’s HSD at α = 0.05. 

To investigate if there were any changes in *H. zea* larval occurrence and ear injury area over time, the relative larval occurrence rate on a *Bt* maize trait in a trial was calculated by dividing the occurrence on the *Bt* trait by the occurrence on the non-*Bt* traits in the trial. The % reduction in ear injury on a *Bt* maize trait in a trial was computed using the formula: % reduction = 100 × (injury area on non-*Bt* ears − injury area on *Bt* ears)/injury area on non-*Bt* area. The data on the relative larval occurrence and % reduction in ear injury after converting with arsine (x)^0.5^ for normality, together with the data of two field trials in a similar study in 2018 [4], were analyzed with a 2-way ANOVA (SAS PROC GLM) [34] with year and *Bt* maize trait as the two main factors and the trial in each year as replication. Furthermore, linear regression (SAS PROC REG) was also used to analyze if there were any trends during the 5-year period of the studies (2018 to 2022) in the relative larval occurrence and % reduction in ear injury for each *Bt* trait. 

## Figures and Tables

**Table 1:** Maize traits and hybrids evaluated in this study.

Maize Trait	Hybrid	Abb. in Figure	*Bt* Toxins for Lepidopteran Pest Species
NBt: Non-*Bt* maize	DKC 65-93	NBt1	Non-*Bt* maize hybrids genetically closely related to one or more *Bt* hybrids used in the study
	DKC 67-70	NBt2	
	DKC 62-05	NBt3	
	DKC 68-24	NBt4	
	DKC 63-56	NBt5	
	DKC 66-94	NBt6	
	DKC 67-25	NBt7	
	DKC 67-25	NBt8	
	NK 1694-GT	NKNBt	
VT2P: Genuity VT Double PRO^®^	DKC 67-72	VT2P1	Cry1A.105, Cry2Ab2
	DKC-67-44	VT2P2	
	DKC 65-95	VT2P3	
	DKC 70-27	VT2P4	
	DKC 66-18	VT2P5	
SMT: Genuity SmartStax^®^	DKC 63-08	SMT1	Cry1A.105, Cry2Ab2, Cry1F
	DKC 67-42	SMT2	
	DKC 62-08	SMT3	
	DKC 65-94	SMT4	
TRE: Trecepta^®^	DKC 65-99	TRE1	Cry1A.105, Cry2Ab2, Cry1F, Vip3Aa20
	DKC 67-94	TRE2	
	DKC 67-99	TRE3	
LEP: Optimum^®^ AcreMax^®^ Leptra™	PI 1622 VYHR	LEP	Cry1Ab, Cry1F, Vip3Aa20
VPT: Agrisure VipteraTM	NK 1694-3111	VPT	Cry1Ab, Vip3Aa20

**Table 2:** Susceptibility of laboratory and field-collected populations of *Helicoverpa zea* from non-*Bt* and two UXI maize plots to three common *Bt* toxins. Ratios for resistance are expressed as positive values, while increased susceptibility compared to the BZ strain is expressed as negative values *.

Insect Population	No. Larvae Assayed	Slope ± SE	LC_50_ (95%CI) (µg/cm^2^)	χ^2^	*p*-Value	Resistance/Susceptibility Ratio
Cry1A.105						
BZ	1152	2.06 ± 0.20	0.014 (0.011, 0.016)	20.7	0.1104	-
NBT_DL_	590	n/a	>>10 (mortality: 12.5%)	n/a	n/a	741
NBT_SG_	1134	0.98 ± 0.12	2.55 (1.80, 4.20)	14.5	0.4131	232
UXI_LA1_	638	1.28 ± 0.30	3.82 (2.27, 7.36)	16.2	0.0927	234
UXI_LA2_	1140	1.02 ± 0.24	1.87 (0.92, 3.78)	64.0	<0.0001	134
Cry2Ab2						
BZ	1148	2.31 ± 0.31	0.126 (0.091, 0.169)	67.5	<0.0001	-
NBT_DL_	589	2.67 ± 0.70	1.56 (0.71, 2.52)	43.3	<0.0001	12
NBT_SG_	1130	1.69 ± 0.20	0.990 (0.752, 1.35)	28.5	0.0121	8
UXI_LA1_	631	1.65 ± 0.34	0.571 (0.267, 0.927)	38.5	0.0004	5
UXI_LA2_	1139	0.94 ± 0.16	0.672 (0.359, 1.16)	66.9	<0.0001	5
Vip3A						
BZ-SS	557	2.23 ± 0.22	0.451 (0.364, 0.559)	11.5	0.8294	-
NBT_DL_	1084	2.72 ± 0.35	0.083 (0.065,0.105)	46.4	0.0003	−5.4
NBT_SG_	1067	1.42 ± 0.09	0.160 (0.133, 0.193)	22.9	0.4066	−2.8
UXI_LA1_	1072	1.26 ± 0.12	0.265 (0.189, 0.364)	49.9	0.0006	−1.7
UXI_LA2_	1139	1.65 ± 0.23	0.016 (0.011, 0.023)	30.7	0.0062	−28.2

* LC_50_ was calculated based on larval practical mortality [6]. The resistance ratio of a field-collected population for the assays against Cry1A.105 and Cry2Ab2 was calculated based on the LC_50_ values of the field-collected population divided by the LC_50_ of BZ, whereas for the bioassays against Vip3A, the susceptibility ratio of a field population was estimated based on the LC_50_ of BZ divided by the LC_50_ of the field-collected populations with a negative sign.

## Data Availability

Data is contained within the article or supplementary material.

## Supplementary Materials

The following supporting information can be downloaded at https://www.mdpi.com/article/10.3390/toxins15070474/s1, Table S1: Field surveys on the larval occurrence of *Helicoverpa zea* and maize ear injury of a non-*Bt* and TRE maize traits in two sentinel plots (SNT-I and SNT-II) near Alexandria, LA, U.S.A. (17 June 2021), Table S2: Establishment of *Helicoverpa zea* lab populations from larvae collected from non-*Bt* maize and sentinel plots with UXIs, Table S3: Occurrence of *Helicoverpa zea* and kernel injury on non-*Bt* and *Bt* corn hybrids in two field trials conducted in 2020 (Trial-I and -II), Table S4: Occurrence of *Helicoverpa zea* and kernel injury on non-*Bt* and Bt corn hybrids in two field trials conducted in 2021 (Trial-III and -IV), Table S5: Occurrence of *Helicoverpa zea* and kernel injury on non-*Bt* and *Bt* corn hybrids in two field trials conducted in 2022 (Trial-V and -VI), Table S6: Kernel injury by *Helicoverpa zea* on non-*Bt* and *Bt* corn hybrids in three additional field trials conducted in 2022 (Trial-VII, -VTIII, and -IX).
