# Supplementary material for: An Extended Investigation of Unexpected Helicoverpa zea (Boddie) Survival and Ear Injury on a Transgenic Maize Hybrid Expressing Cry1A/Cry2A/Vip3A Toxins
Source: Toxins (Basel). 2023 Jul 22;15(7):474. doi: 10.3390/toxins15070474 (PMC10467152; doi:10.3390/toxins15070474)
Supplement: Supplementary file 1 [file toxins-15-00474-s001.zip › toxins-2491323-supplementary.pdf]

toxins MDPI

Supplementary Materials: An Extended Investigation of Unexpected *Helicoverpa zea* (Boddie) Survival and Ear Injury on a Transgenic Maize Hybrid Expressing Cry1A/Cry2A/Vip3A Toxins

Fangneng Huang, Ying Niu, Tiago Silva, Sebe Brown, Tyler Towles, Dawson Kerns, Juan Luis Jurat-Fuentes, Graham P. Head, Matthew Carroll, Wade Walker and Shucong Lin

Table S1. Field surveys on larval occurrence of *Helicoverpa zea* and maize ear injury of a non-Bt and TRE maize traits in two sentinel plots (SNT-I and SNT-II) near Alexandria, LA, USA (June 17, 2021).

SNT plots	GPS	Hybrid	Trait	No. ears sampled	No. ears injured (% injured)	No. ears containing live larvae (%)	No. live larvae (2nd, 3rd, 4th, 5th, 6th instar)	Ear injury area (cm^2/injured ear)
SNT-I	31°10′11.7″N 92°24′42.6″W	DKC 65-99	TRE	1,663	42 (2.5%)	35 (2.1%)	46 (17, 26, 3, 0, 0)	2.3
SNT-II	31°10′27.9″N 92°24′01.2″W	DKC 65-99	TRE	2,442	91 (3.7%)	85 (3.5%)	89 (28, 53, 8, 0, 0)	2.2
SNT-I	31°10′11.7′N 92°24′42.6″W	DKC 65-93	NBt	61	38 (62.3%)	16 (26.2%)	16 (6, 3, 3, 3, 1)	3.8

Table S2. Establishment of *Helicoverpa zea* lab populations from larvae collected from non-Bt maize and sentinel plots with UXIs.

Insect population	Collection date	Location collected	Host plant	No. individuals used to establish lab population	Larval stages collected‡	Generation assayed
BZ-SS	---	Lab	Diet	---	---	---
NBt_{DL}	June 17, 2021	Alexandria, LA	DKC 65-93	87 larvae	3rd - 6th instar larvae	F2
NBt_{SG}	Aug 18, 2021	St. Gabriel, LA	DKC 65-93	132 larvae	3rd - 5th instar larvae	F1
UXI_{LA1}	June 17, 2021	Alexandria, LA	DKC 65-99	55 pupae	3rd - 4th instar larvae	F2
UXI_{LA2}	June 17, 2021	Alexandria, LA	DKC 65-99	63 pupae	3rd - 4th instar larvae	F2

‡ 2nd instars of UXI_{LA1} and UXI_{LA2} collected from field continued to be reared on detached ears in the laboratory until 3rd to 4th instars.

Table S3. Occurrence of *Helicoverpa zea* and kernel injury on non-Bt and Bt corn hybrids in two field trials conducted in 2020 (Trial-I and -II).

Hybrids	Abb.	Winnsboro (Trial-II) No larvae/plant	Winnsboro (Trial-II) Larval development index	Winnsboro (Trial-II) Ear damage	Dean Lee (Trial-I) No larvae/plant	Dean Lee (Trial-I) Larval development index	Dean Lee (Trial-I) Ear damage
DKC 62-05	NBt3	1.46 ± 0.11 bc	4.4 ± 0.4 b	10.5 ± 1.3 d	1.74 ± 0.11 c	4.3 ± 0.4 c	12.3 ± 1.4 d
DKC 68-24	NBt4	1.75 ± 0.09 c	4.5 ± 0.3 b	12.3 ± 1.0 d	1.56 ± 0.11bc	4.4 ± 0.1 bc	11.8 ± 1.1 d
NK-NBt	NKNBt	1.99 ± 0.19 c	3.1 ± 0.5 a	7.0 ± 0.6 cd	n/a	n/a	n/a
DKC 70-27	VT2P4	1.00 ± 0.20 b	2.4 ± 0.0 a	2.6 ± 0.6 b	1.26 ± 0.12 b	2.9 ± 0.2 a	3.9 ± 0.4 b
DKC 66-18	VT2P5	1.53 ± 0.32 bc	2.8 ± 0.1 a	4.9 ± 1.0 bc	1.36 ± 0.05 bc	2.9 ± 0.2 a	3.7 ± 0.3 b
DKC 63-08	SMT1	1.68 ± 0.16 c	3.3 ± 0.2 a	9.1 ± 0.6 d	1.75 ± 0.18 c	3.1 ± 0.1 a	5.7 ± 0.5 c
DKC 67-42	SMT2	1.40 ± 0.14 bc	2.7 ± 0.2 a	4.2 ± 0.5 bc	1.56 ± 0.11 bc	2.8 ± 0.1 a	3.7 ± 0.3 b
DKC 65-99	TRE1	0.00 ± 0.00 a	n/a	0.0 ± 0.0 a	0.00 ± 0.00 a	n/a	0.0 ± 0.0 c
DKC 67-99	TRE3	0.01 ± 0.01 a	4 *	0.1 ± 0.1 a	0.00 ± 0.00 a	n/a	0.0 ± 0.0 c
NK 1694-3111	VPT	0.00 ± 0.00 a	n/a	0.0 ± 0.0 a	0.00 ± 0.00 a	n/a	0.0 ± 0.0 c
ANOVA		$F_{9,25} = 51.62$ $P < 0.0001$	$F_{6,16} = 14.41$ $P < 0.0001$	$F_{9,25} = 91.94$ $P < 0.0001$	$F_{8,24} = 139.53$ $P < 0.0001$	$F_{5,15} = 18.48$ $P < 0.0001$	$F_{8,24} = 260.86$ $P < 0.0001$

Mean values within a column followed by the same letter are not significantly different (Tukey HSD test, $\alpha = 0.05$).

Table S4. Occurrence of *Helicoverpa zea* and kernel injury on non-Bt and Bt corn hybrids in two field trials conducted in 2021 (Trial-III and -IV).

Hybrids	Abb.	Winnsboro (Trial-IV) No larvae/plant	Winnsboro (Trial-IV) Larval development index	Winnsboro (Trial-IV) Ear damage	Dean (Trial-III) No larvae/plant	Dean (Trial-III) Larval development index	Dean (Trial-III) Ear damage
DKC 65-93	NBt1	0.50 ± 0.08 b	4.9 ± 0.2 bc	5.1 ± 0.3 bc	1.39 ± 0.13 bcd	5.7 ± 0.1 ab	13.2 ± 1.2 cd
DKC 67-70	NBt2	0.65 ± 0.07 b	5.0 ± 0.1 bc	6.1 ± 0.4 cd	1.50 ± 0.17 cd	5.9 ± 0.0 b	15.3 ± 2.1 d
NKNBT	NKNBt	1.03 ± 0.05 c	5.2 ± 0.1 c	9.2 ± 0.6 d	1.89 ± 0.01 d	5.9 ± 0.0 b	20.0 ± 1.1 d
DKC 67-72/67-44	VT2P1/VT2P2	1.30 ± 0.15 c	4.4 ± 0.2 ab	6.9 ± 0.4 d	0.88 ± 0.09 bc	4.9 ± 0.4 ab	7.5 ± 1.4 b
DKC 66-18/65-95	VT2P5/VT2P3	1.19 ± 0.07 c	3.9 ± 0.1 a	4.6 ± 0.5 b	1.04 ± 0.15 b	4.9± 0.3 ab	8.4 ± 1.1bc
DKC 67-42	SMT2	1.08 ± 0.09 c	4.1 ± 0.2 a	5.4 ± 0.4 bcd	0.94 ± 0.16 b	4.5 ± 0.3 a	7.2 ± 1.1 b
DKC 62-08/65-94	SMT3/SMT4	1.06 ± 0.03 c	4.4 ± 0.1 ab	6.3 ± 0.4 cd	0.88 ± 0.12 b	5.2 ± 0.2 ab	8.3 ± 0.9 bc
DKC 65-99	TRE1	0.00 ± 0.00 a	n/a	0.0 ± 0.0 a	0.01 ± 0.01 a	4*	0.1 ± 0.1 a
DKC-67-94	TRE2	0.00 ± 0.00 a	n/a	0.0 ± 0.0 a	0.00 ± 0.00 a	n/a	0.0 ± 0.0 a
NK 1694-3111	VPT	0.01 ± 0.01 a	4*	0.0 ± 0.0 a	0.00 ± 0.00 a	n/a	0.0 ± 0.0 a
PI 1622 VYHR	LEP	0.00 ± 0.00 a	n/a	0.0 ± 0.0 a	0.00 ± 0.00 a	n/a	0.0 ± 0.0 a
ANOVA		$F_{10,30} = 114.78$ $P < 0.0001$	$F_{6,18} = 12.38$ $P < 0.0001$	$F_{10,30} = 362.64$ $P < 0.0001$	$F_{10,30} = 64.75$ $P < 0.0001$	$F_{6,18} = 3.80$ $P = 0.0127$	$F_{10,30} = 175.91$ $P < 0.0001$

Mean values within a column followed by the same letter are not significantly different (Tukey HSD test, $\alpha = 0.05$).

Table S5. Occurrence of *Helicoverpa zea* and kernel injury on non-Bt and Bt corn hybrids in two field trials conducted in 2022 (Trial-V and -VI).

Hybrids	Abb.	Winnsboro (Trial-VI)			Dean Lee (Trial-V)		
		No larvae/plant	Larval developmental index	Ear damage	No larvae/plant	Larval developmental index	Ear damage
DKC 65-93	NBt1	0.25 ± 0.06 bc	5.6 ± 0.3 a	3.7 ± 0.3 bc	1.54 ± 0.13 b	3.7 ± 0.2 b	8.0 ± 0.3 cd
DKC 67-70	NBt2	0.40 ± 0.11 c	4.8 ± 0.9 a	5.3 ± 0.4 d	1.76 ± 0.10 bc	4.8 ± 0.1 c	14.7 ± 0.8 d
NKNBT	NKNBt	0.44 ± 0.08 c	5.4 ± 0.1 a	4.5 ± 0.5 cd	1.91 ± 0.06 bcd	4.5 ± 0.2 c	14.4 ± 2.1 d
DKC 67-72	VT2P1	0.25 ± 0.07 bc	3.9 ± 0.6 a	3.2 ± 0.3 bc	2.51 ± 0.26 d	3.1 ± 0.1 ab	7.5 ± 1.8 bc
DKC 65-95	VT2P3	0.33 ± 0.06 c	4.2 ± 0.4 a	3.1 ± 0.2 bc	2.16 ± 0.08 cd	3.0 ± 0.1 a	3.9 ± 0.7 b
DKC 67-42	SMT2	0.44 ± 0.02 c	4.4 ± 0.4 a	4.0 ± 0.4 bcd	3.20 ± 0.16 e	3.3 ± 0.1 ab	9.4 ± 1.0 cd
DKC 65-94	SMT4	0.20 ± 0.05 abc	4.3 ± 0.4 a	2.8 ± 0.3 b	2.21 ± 0.12 cd	3.2 ± 0.2 ab	7.0 ± 1.5 bc
DKC 65-99	TRE1	0.00 ± 0.00 a	N/A	0.0 ± 0.0 a	0.03 ± 0.01 a	3.0±0.0 (2 3rd)*	0.0 ± 0.0 a
DKC-67-94	TRE2	0.01 ± 0.01 ab	4*	0.0 ± 0.0 a	0.00 ± 0.00 a	n/a	0.0 ± 0.0 a
NK 1694-3111	VPT	0.00 ± 0.00 a	N/A	0.0 ± 0.0 a	0.01 ± 0.01 a	2.0 (1 2nd) *	0.0 ± 0.0 a
PI 1622 VYHR	LEP	0.00 ± 0.00 a	N/A	0.0 ± 0.0 a	0.03 ± 0.01 a	3.5 ± 0.5 (3rd, 4th)*	0.1 ± 0.1 a
ANOVA		$F_{10,30} = 12.04$ $P < 0.0001$	$F_{6,18} = 1.33$ $P = 0.2928$	$F_{10,30} = 179.8$ $P < 0.0001$	$F_{10,30} = 242.64$ $P < 0.0001$	$F_{6,18} = 21.88$ $P = 0.0001$	$F_{10,30} = 103.86$ $P < 0.0001$

Mean values within a column followed by the same letter are not significantly different (Tukey HSD test, $\alpha = 0.05$).

Table S6. Kernel injury by *Helicoverpa zea* on non-Bt and Bt corn hybrids in three additional field trials conducted in 2022 (Trial-VII, -VTIII, and -IX).

Hybrids	Abb.	St. Joseph (Trial-IX)	Dee Lee (Trial-VII)	Winnsboro (Trial-VIII)
DKC 65-93	NBt1	6.4 ± 0.7 bc	10.1 ± 1.4 b	4.3 ± 0.4 bc
DKC 67-70	NBt2	9.4 ± 0.8 de	16.7 ± 1.5 bc	3.8 ± 0.4 bc
NKNBT	NKNBt	10.4 ± 0.6 e	19.7 ± 1.5 c	4.3 ± 0.1 bc
DKC 62-05	NBt3	8.0 ± 0.7 bcde	13.8 ± 1.9 bc	4.7 ± 0.7 c
DKC 63-56	NBt5	8.8 ± 0.7 cde	10.0 ± 0.9 b	5.7 ± 0.8 c
DKC 66-94	NBt6	6.8 ± 0.5 bcd	11.5 ± 1.0 bc	4.0 ± 0.2 bc
DKC 70-25	NBt7	9.2 ± 0.7 de	14.3 ± 1.4 bc	3.0 ± 0.5 b
DKC-67-72	VT2P1	5.9 ± 0.4 b	13.2 ± 0.9 bc	0.3 ± 0.1 a
DKC 67-94	TRE2	0.0 ± 0.0 a	0.3 ± 0.2 a	0.0 ± 0.0 a
NK 1694-3111	VPT	0.0 ± 0.0 a	0.7 ± 0.4 a	0.0 ± 0.0 a
PI 1622 VYHR	LEP	0.0 ± 0.0 a	0.1 ± 0.1 a	0.0 ± 0.0 a
ANOVA		$F_{10,30} = 276.06$ $P < 0.0001$	$F_{10,30} = 91.36$ $P < 0.0001$	$F_{10,30} = 133.73$ $P < 0.0001$

Mean values within a column followed by the same letter are not significantly different (Tukey HSD test, $\alpha = 0.05$).
